# Limitation to Advanced Life Support in patients admitted to intensive care unit with integrated palliative care

**DOI:** 10.5935/0103-507X.20160042

**Published:** 2016

**Authors:** Sandra Regina Gonzaga Mazutti, Andréia de Fátima Nascimento, Renata Rego Lins Fumis

**Affiliations:** 1 Intensive Care Unit, Hospital Paulistano - São Paulo (SP), Brazil.; 2 Department of Public Health, Faculdade de Ciências Médicas, Santa Casa de São Paulo - São Paulo (SP), Brazil.; 3 Intensive Care Unit, Hospital Sírio-Libanês - São Paulo (SP), Brazil.

**Keywords:** Life support care, Palliative care, Intensive care units

## Abstract

**Objective:**

To estimate the incidence of limitations to Advanced Life Support in critically ill patients admitted to an intensive care unit with integrated palliative care.

**Methods:**

This retrospective cohort study included patients in the palliative care program of the intensive care unit of *Hospital Paulistano* over 18 years of age from May 1, 2011, to January 31, 2014. The limitations to Advanced Life Support that were analyzed included do-not-resuscitate orders, mechanical ventilation, dialysis and vasoactive drugs. Central tendency measures were calculated for quantitative variables. The chi-squared test was used to compare the characteristics of patients with or without limits to Advanced Life Support, and the Wilcoxon test was used to compare length of stay after Advanced Life Support. Confidence intervals reflecting p ≤ 0.05 were considered for statistical significance.

**Results:**

A total of 3,487 patients were admitted to the intensive care unit, of whom 342 were included in the palliative care program. It was observed that after entering the palliative care program, it took a median of 2 (1 - 4) days for death to occur in the intensive care unit and 4 (2 - 11) days for hospital death to occur. Many of the limitations to Advanced Life Support (42.7%) took place on the first day of hospitalization. Cardiopulmonary resuscitation (96.8%) and ventilatory support (73.6%) were the most adopted limitations.

**Conclusion:**

The contribution of palliative care integrated into the intensive care unit was important for the practice of orthothanasia, i.e., the non-extension of the life of a critically ill patient by artificial means.

## Abstract (pt)

**Objetivo:**

Estimar a incidência de limitação de Suporte Avançado de Vida em pacientes graves internados em unidade de terapia intensiva com cuidados paliativos integrados.

**Métodos:**

Estudo de coorte retrospectivo, no qual foram incluídos os pacientes inseridos no programa de cuidados paliativos da unidade de terapia intensiva do Hospital Paulistano, maiores de 18 anos de idade, no período de 1º de maio de 2011 a 31 de janeiro de 2014. As limitações de Suporte Avançado de Vida analisadas foram: ordem para não ressuscitar, ventilação mecânica, hemodiálise e droga vasoativa. Para as variáveis quantitativas, foram calculadas medidas de tendência central. O teste qui quadrado foi utilizado para comparar características dos pacientes com ou sem limitação de Suporte Avançado de Vida e teste de Wilcoxon, para comparar o tempo de internação após Suporte Avançado de Vida. Para significância estatística, consideraram-se o intervalo de confiança e p ≤ 0,05.

**Resultados:**

Foram internados na unidade de terapia intensiva 3.487 pacientes, sendo 342 inseridos no programa de cuidados paliativos. Observou-se que, após entrada no programa de cuidados paliativos, demorou uma mediana de 2 (1 - 4) dias para o óbito na unidade de terapia intensiva e 4 (2 - 11) dias para óbito hospitalar. Boa parte das limitações de Suporte Avançado de Vida (42,7%) aconteceu no primeiro dia de internação. A ressuscitação cardiopulmonar (96,8%) e o suporte ventilatório (73,6%) foram as limitações mais adotadas.

**Conclusão:**

Foi relevante a contribuição dos cuidados paliativos integrados à unidade de terapia intensiva para a prática da ortotanásia, ou seja, o não prolongamento da vida de um paciente terminal por meios artificiais.

## INTRODUCTION

Many intensive care unit (ICU) admissions do not justify use of the high level of technology and human resources available and ultimately keep critically ill patients with advanced diseases or who are terminally ill alive for a prolonged period of time, without at least knowing their treatment preferences.^(1)^

The process of dying is often prolonged, accompanied by aggressive interventions, without adopting comfort measures.^(2)^ Many survivors remain in a chronic critical condition, with severe functional and cognitive impairment - a scenario that has been observed in several countries.^(3)^

Currently, there is a growing trend toward allowing dying with dignity, instead of pointlessly prolonging the life and suffering of both the patient and family with futile treatments.^(4)^ Palliative care is prioritized in order to give more quality to the time the patient remains alive, with measures to promote physical, emotional, social and spiritual comfort. Therefore, in the case of critically ill patients, even those already in the ICU, the integration of palliative care is of paramount importance and brings numerous benefits.^(5-8)^

Laws governing the patients' right to anticipate their decisions on care procedures, that is, advance directives, have been created and reiterated in countries in North America, South America and Europe.^(8-12)^

In Brazil, the Federal Council of Medicine (Conselho Federal de Medicina - CFM), through Resolution 1,805/2006, decided that the physician is allowed to limit or suspend procedures and treatments that prolong the life of patients in the terminal phase of a critical and incurable illness, thereby respecting the wishes of the person or his/her legal representative and consequently basing the medical decision on the philosophy of palliative care.^(10)^ The patient's right to express his or her wishes has been guaranteed through advance directives in resolution 1,955/2012 of the CFM.^(11)^

Brazilian studies provide evidence that Brazilian society is changing and that Brazilians are discussing this practice.^(13-17)^ Moritz et al. demonstrated that the refusal or suspension of treatment was observed in 32% of deaths in the ICU of a university hospital and that the futility of treatment was considered the main reason for doing so in 100% of these cases.^(14)^ In Bitencourt et al.'s study, for 35.8% of patients who died in the ICU, treatment limitation was associated to a length of stay of more than 10 days.^(15)^ In a more recent study, Moritz et al. observed that disease terminality had been recognized in 40.0% of ICU cases and that treatment refusal/suspension preceded 30.7% of deaths, after patients had been in the ICU for an average of 8.7 days.^(16)^

Despite these studies, the frequency with which Advanced Life Support (ALS) is limited is still low, and the time spent in the ICU until the occurrence of death is still very long compared with other countries.^(18,19)^ Brazil is one of the lowest-ranked countries (38 of 40) in terms of quality of dying, according to a study conducted by Economist Intelligence.^(20)^

There are few studies in Brazil on the practice of limiting ALS in patients with terminal illness, especially in ICUs with integrated palliative care. This study aimed to estimate the frequency with which ALS is not initiated among critically ill patients in the intensive care unit of a private hospital with integrated palliative care.

## METHODS

This retrospective cohort study was conducted with a consecutive sample of patients over 18 years of age admitted to the ICU of *Hospital Paulistano*, with treatment information in their medical records, during the period from May 1, 2011, to January 31, 2014. The data source was the *Hospital Paulistano* Palliative Care Service's database. The study was approved by the Research Ethics Committee of *Hospital Sírio-Libanês*, under number HSL 2014-45.

The *Hospital Paulistano* is a private hospital in the Amil network, with an ICU of 20 private beds and with an open visiting policy from 8:00 am to 8:00 pm. Upon admission, on the hospital consent form, the patients were already informed about the palliative care program and how they could integrate it if they had a chronic progressive advanced critical illness and low functional status. Patients and their families were informed of the prognosis, progress and treatment plan before joining the program.

To calculate the frequency with which ALS was limited, those patients enrolled in the palliative care program prior to the introduction of ALS were considered.

The data regarding limitations to ALS were collected by the palliative care nurse directly from the patient medical records, as filled out by the medical team. During this collection time, a guideline checklist was not used; thus, progression was recorded in the each physician's own words. The data extracted from the medical records were transferred to the "data registration form" specific to the palliative care team and compiled in an Excel spreadsheet by the same palliative care nurse.

The following variables were analyzed: age, gender, baseline diagnosis, sedation, Simplified Acute Physiology Score (SAPS 3), Karnofsky Performance Status (KPS) and limitation to ALS, with respect to limitations in the use of mechanical ventilation, dialysis and vasoactive drugs. The 40% cutoff point (corresponding to a state of incapacity in which the patient needs special care and assistance) was used for patient performance classification, according to the KPS. A cutoff score of 49 points was used for the SAPS 3, which the literature supports as representing greater severity.

### Statistical analysis

Absolute and relative frequencies were calculated for qualitative variables. For quantitative variables, central tendency (mean or median) and dispersion (standard deviation - SD, or first - p_25_ and third - p_75_ quartiles) measures were employed. The incidences of limiting ALS measures and their respective confidence intervals (95%CI) were calculated.

The chi-squared test was used to compare the characteristics of patients with or without ALS limitations. The Wilcoxon test was used to compare the lengths of stay in the ICU and in the hospital, according to the indication for ALS limitations. The level of statistical significance used was p ≤ 0.05. Statistical analysis was performed using Stata^®^ software (version 11.1)

## RESULTS

A total of 3,487 patients were admitted to the ICU in the period from May 1, 2011, to January 31, 2014. The characteristics of the patients admitted to the ICU during the study period are shown in table 1.

**Table 1:** Demographic and clinical characteristics of patients admitted to the intensive care unit

Characteristics	Results N = 3,487
Female	1,789 (51.3)
Age (years)	62.8 ± 18.7
Clinical diagnoses	2,598 (74.5)
Karnofsky Performance Status	64.7 ± 24.4
Simplified Acute Physiology Score 3	43.2 ± 15.2
Length of ICU stay (days)	2 (1 - 4)
Length of hospital stay (days)	10 (5 - 19)
ICU mortality	352 (10.1)
Hospital mortality	634 (18.2)

ICU - intensive care unit. Results are expressed as number (%), mean ± standard deviation and median (25% - 75%).

The length of the ICU stay ranged from 1 to 58 days, with a median of 2 (1 - 4) days; 112 (3.2%) patients stayed in the ICU for a period of ≥ 15 days. The length of stay in the hospital ranged from 1 to 418 days, with a median of 10 (5 - 19) days, and 304 (8.7%) patients stayed in the hospital for a period of ≥ 40 days.

A total of 352 (10.1%) patients died during their stays in the ICU and 634 (18.2%) during their stay in the hospital (Table 1). Regarding the palliative care program, 342 patients were included in that program, corresponding to an incidence of 9.8% (95%CI: 8.8% - 10.8%). Inclusion in the palliative care program was associated with older age, clinical diagnosis, a KPS score of ≤ 40% and a SAPS 3 score ≥ 49 points (p < 0.001) (Table 2).

**Table 2:** Demographic and clinical characteristics of patients admitted to the intensive care unit, according to inclusion or non-inclusion in the palliative care program (N = 3,487)

Variables	Inclusion in the palliative care program		
	No (N = 3,145) N (%)	Yes (N = 342) N (%)	p value*
Sex			0.123
Female	1,600 (89.4)	189 (10.6)	
Male	1,545 (91.0)	153 (9.0)	
Age range (years)			< 0.001
Up to 39	476 (96.4)	18 (3.6)	
40 - 59	872 (95.7)	39 (4.3)	
60 - 79	1,233 (90.3)	131 (9.7)	
≥ 80	574 (78.8)	154 (21.2)	
Type of admission			< 0.001
Clinical	2,299 (73.2)	299 (87.4)	
Surgical	842 (26.8)	43 (12.6)	
Karnofsky Performance Status			< 0.001
≤ 40%	337 (55.7)	268 (44.3)	
> 40%	2,808 (97.4)	74 (2.3)	
Simplified Acute Physiology Score 3			< 0.001
< 49	2,203 (96.7)	74 (2.3)	
≥ 49	942 (77.8)	268 (44.3)	

* p = chi-square test.

The main indications for inclusion in the palliative care program were cancer diagnosis (139; 40.6%), followed by chronic organ failure (55; 16.1%).

The adoption of an ALS limitation occurred between the first day of admission to the ICU and several days thereafter, with a median of 2 (1 - 5) days; 146 (42.7%) patients adopted a limitation of ALS on the first day of hospitalization (Table 3). The most-adopted ALS limitation measures were cardiopulmonary resuscitation (331; 96.8%) and the instruction not to undergo invasive mechanical ventilation (251; 73.6%). Moreover, 113 (33.0%) patients received palliative sedation.

**Table 3:** Advanced Life Support limitations during the intensive care unit stays of patients in the palliative care program

Variables	Results N = 342
Time to indication of an ALS limitation in the ICU (days)	2 (1 - 5)
Cardiopulmonary resuscitation limitation	
Yes	331 (96.8)
Orotracheal intubation limitation	
Yes	252 (73.6)
Renal replacement therapy limitation	
Yes	235 (68.7)
Vasoactive drugs limitation	
Yes	191 (55.8)
Palliative sedation	
Yes	113 (33.0)

ALS - Advanced life support; ICU - intensive care unit. Results are expressed as number (%), mean ± standard deviation and median (25% - 75%).

Patients included in the palliative care program had longer hospital and ICU stays than those who did not have this condition (p < 0.001) (Table 4). Among the 342 included in the palliative care program, 115 (33.6%) died during their ICU stays, whereas among the 3,145 patients who were not included in the program, 237 (7.5%) died in the ICU (p < 0.001). Throughout their hospital stays, the mortality rates of patients included or not included in the palliative care program were 73.1% and 18.2%, respectively (p < 0.001). It was observed that there was a median of 2 (1 - 4) days from initial inclusion in the palliative care program to death in the ICU and of 4 (2 - 11) days to hospital death, with a significant difference in discharge time (p < 0.001 for both outcomes; Table 5).

**Table 4:** Lengths of stay in the intensive care unit and the hospital, according to inclusion or non-inclusion in the palliative care program

Variables	N	Median	p_25_-p_75_	p value*
Length of hospital stay (days)				
Palliative care program				
No	3,145	9	5 - 18	
Yes	342	16	8 - 31	< 0.001
Length of ICU stay (days)				
Palliative care program				
No	3,145	2	1 - 3	
Yes	342	4	2 - 7	< 0.001

ICU - intensive care unit.

* p = Wilcoxon test.

**Table 5:** Lengths of stay in the intensive care unit and the hospital for patients included in the palliative care program, according to outcome of stay (N = 342)

	N	Median	p_25_-p_75_	p value*
Length of hospital stay (days)				< 0.001
Discharge	92	17	10 - 27	
Death	250	4	2 - 11	
Length of ICU stay (days)				< 0.001
Discharge	227	1	0 - 2	
Death	115	2	1 - 4	

ICU - intensive care unit.

* p = Wilcoxon test.

## DISCUSSION

This study described the end-of-life care of critically ill patients in an ICU with integrated palliative care, with an emphasis on non-adoption of ALS measures, such as intubation, hemodialysis, use of vasoactive drugs and do-not-resuscitate orders, which potentially prolong the lives of patients with poor prognoses.

Although a paternalistic culture is still prevalent in Brazil,^(21)^ communication of final decisions is part of routine integrated palliative care in *Hospital Paulistano*'s ICU. Decisions are shared with all family members and, when possible, include the participation of patients in the palliative program who decide on ALS limitations.

Effective communication is a factor that is essential for success in this type of decision-making. A conversation with the family should be held immediately at the beginning of the first week of hospitalization. Effective communication with the patient and family about the treatment options, preferably within the first 72 hours of ICU admission, facilitates the decision-making process.^(22)^

In the ICU herein studied, the palliative care team confers with the family at the beginning of hospitalization, which may have allowed decisions on treatment limitations in the ICU to be made earlier.

However, it was not possible to identify how many patients actually participated in the discussion on ALS limitations with the team because during the period in which the study was conducted, there was no record of patient participation, although the family participated in all decisions.

According to this study, the integration of palliative care into the ICU can help reduce the time required to determine treatment goals that are in keeping with the wishes and values of the patient and family. This study showed that in most cases, the decision to limit ALS measures occurred on the first day, whereas in the literature, a longer delay is normally observed, ranging from 4 to 7 days.^(19,23)^

In Brazil, the length of stay in the ICU until the occurrence of death is considered too long compared to international studies, possibly due to the lack of adoption of treatment limits. Clinical treatment and a length of ICU stay greater than 10 days are independent factors that are associated with measures indicative of adopting treatment limitations.^(15)^

In this study, the time from inclusion in the palliative care program until death in the ICU was a median of 2 (1 - 4) days, whereas a median of 4 (2 - 11) days was observed for hospital death. Those values are much lower than those observed in the literature, where the reported time to death is 5 (2 - 13) days in the ICU after establishing ALS limitations.^(18)^

It is known that some factors are very closely associated with the decision to limit ALS, such as advanced age, cognitive impairment, disease severity, previous comorbidities and having limited quality of life, in addition to the wishes and preferences of patients and families.^(18,19)^

Most patients with ALS limitations are of an advanced age (over 70) and have low KPS scores and high SAPS 3 scores (> 49 points), per typical observations from the literature.^(18)^

Considering the aging population, particularly in a country with a demographic profile such as Brazil, and consequent increases in the frequency of chronic diseases, the subject of palliative care should be further explored with regard to its application in ICUs.^(24)^

In addition to these factors, patients who have indications for ALS limitations were more often those with advanced cancer and chronic organ failure. Although this study was performed in an institution that is a referral hospital for the treatment of cancer patients, the differentiator enabling these patients not to undergo futile treatment, even with advanced cancer, was an integrated palliative care service. It is part of the ICU's routine to monitor for cases eligible for palliative care and to discuss this option with family members. These decisions are shared from the time of the patient's admission to the ICU.

A recent national study performed in another major cancer center, but without palliative care in the ICU, showed that for patients with advanced cancer, the lack of indication for palliative care was significantly associated with futile treatment in the ICU.^(17)^

Curative medicine in the ICU helps to prolong life but neglects to provide end-of-life quality. Palliative medicine is therefore important for bringing general medicine closer to human values and dignity.^(25)^

It is interesting to note that patients who entered the palliative care program and who had ALS limitations had longer hospital and ICU stays than those who did not have this condition, with higher mortalities in the ICU and during their hospital stays. Undoubtedly, longer hospitalization is associated with disease severity and comorbidities present, but it is of the utmost importance to note that after making treatment limitation decisions, the majority (73.3%) died outside of the ICU. Therefore, integrated palliative care in the ICU can provide many benefits, as seen in this study: almost 100% of patients who entered the program were spared futile treatment and could be closer to their families in their final moments, providing them with more dignity and a shorter time to death. It is important for integration of palliative care to take place earlier so that these patients can die, if possible, at home, where a better quality of life is reported.^(26)^ Dying in the ICU or hospital, even with integrated palliative care, is still a sign of greater discomfort, pain and anxiety and poorer quality of life.^(27)^

Understanding expectations and identifying patient and family preferences are part of the process. It is therefore a very complex subject that depends on, among other things, cultural factors. Interestingly, there is great variability in final decisions regarding whether to institute or discontinue ALS treatment across countries around the world and across hospitals, ICUs and physicians.^(4)^

This study identified that cardiopulmonary resuscitation (CPR) in the ICU was limited in almost 100% of cases. This finding is in line with the literature, which shows that it is very common to have this order written in the medical records of patients with terminal prognoses.^(28)^

Ethicists argue that CPR should not be administered to patients with no chance of benefiting from it.^(29)^ Our findings are consistent with other Brazilian studies in which CPR is the most common ICU limitation, followed by vasoactive drugs and dialysis.^(15,16)^

It is noteworthy that ALS limitations, which were frequently observed in this study, occurred for cases in which patients had not yet undergone intubation. One of the limitations of this study was that we did not include the withdrawal of life support, but we know that withdrawal of the ventilator is rare in Brazil.^(15)^

Treatment changes toward palliative care allowed the patients the opportunity to die in their rooms alongside their families in a more humanistic way or even in the ICU, but in a dignified manner. One third of the patients received palliative sedation, which is a justifiable procedure from a legal ethical point of view and which aims to relieve intolerable and treatment-refractory symptoms, reduce pain, provide comfort and relieve the patient of intolerable distress. This measure is widely recognized as the appropriate approach to end-of-life care and should not be confused with euthanasia, which aims to hasten death; instead, palliative sedation is intended to bring comfort and dignity to the patient and family.^(30)^

This study had some limitations. First, it had a retrospective design; therefore, it was not possible to perform a comparative analysis among patients admitted to the ICU but who did not enter the palliative care program for the purpose of determining whether they had ALS limitations.

It was also a single-center study and one with very specific characteristics regarding care, so it is not possible to generalize the data.

Another limitation was the failure to identify reasons for the ICU admission, which are important data for analysis. Typically, patients with advanced diseases are admitted with acute respiratory failure, sepsis, renal failure and reduction in consciousness level, among other things. Nonetheless, clinical admission was the most indicated outcome for establishing ALS limitations.

## CONCLUSION

The issue addressed in the present study was the practice of orthothanasia, or the non-prolonging of a terminally ill patient's life by artificial means. The contribution of integrated palliative care, so that these decisions could be made in the intensive care unit, was evident. However, more multicenter studies are required to better explore these issues and understand the current situation in Brazil in order to confirm the importance of early palliative care in patient care.
